# Abrogation of Notch Signaling in Embryonic TECs Impacts Postnatal mTEC Homeostasis and Thymic Involution

**DOI:** 10.3389/fimmu.2022.867302

**Published:** 2022-05-30

**Authors:** María Jesús García-León, Marta Mosquera, Carmela Cela, Juan Alcain, Saulius Zuklys, Georg Holländer, María L. Toribio

**Affiliations:** ^1^ Immune System Development and Function Unit, Centro de Biología Molecular Severo Ochoa, Consejo Superior de Investigaciones Científicas (CSIC), Universidad Autónoma de Madrid (UAM), Madrid, Spain; ^2^ Department of Biomedicine and University Children’s Hospital of Basel, University of Basel, Basel, Switzerland; ^3^ Department of Paediatrics and the Weatherall Institute of Molecular Medicine, University of Oxford, Oxford, United Kingdom

**Keywords:** thymus, notch, thymic epithelial cells, premature degeneration, thymic involution

## Abstract

Notch signaling is crucial for fate specification and maturation of thymus-seeding progenitors along the T-cell lineage. Recent studies have extended the role of Notch signaling to thymic epithelial cells (TECs), showing that Notch regulates TEC progenitor maintenance and emergence of medullary TECs (mTECs) in fetal thymopoiesis. Based on immunohistochemistry studies of spatiotemporal regulation of Notch activation in the postnatal thymus, we show that *in vivo* Notch activation is not confined to fetal TECs. Rather, Notch signaling, likely mediated through the Notch1 receptor, is induced in postnatal cortical and medullary TECs, and increases significantly with age in the latter, in both humans and mice, suggesting a conserved role for Notch signaling in TEC homeostasis during thymus aging. To investigate the functional impact of Notch activation in postnatal TEC biology, we used a mouse model in which RPBJκ, the transcriptional effector of canonical Notch signaling, is deleted in epithelial cells, including TECs, under the control of the transcription factor Foxn1. Immunohistochemistry and flow cytometry analyses revealed no significant differences in TEC composition in mutant (RPBJκ-KO^TEC^) and wild-type (WT) littermate mice at early postnatal ages. However, a significant reduction of the medullary region was observed in mutant compared to WT older thymi, which was accompanied by an accelerated decrease of postnatal mTEC numbers. Also, we found that organization and integrity of the postnatal thymic medulla critically depends on activation of the canonical Notch signaling pathway, as abrogation of Notch signaling in TECs led to the disruption of the medullary thymic microenvironment and to an accelerated thymus atrophy. These features paralleled a significant increase in the proportion of intrathymic non-T lineage cells, mostly B cells, and a slight decrease of DP thymocyte numbers compatible with a compromised thymic function in mutant mice. Therefore, impaired Notch signaling induced in embryonic development impacts postnatal TECs and leads to an accelerated mTEC degeneration and a premature thymus involution. Collectively, our data have uncovered a new role for Notch1 signaling in the control of adult mTEC homeostasis, and point toward Notch signaling manipulation as a novel strategy for thymus regeneration and functional recovery from immunosenescence.

## Introduction

T lymphocytes, unlike the rest of blood cell lineages derived from multipotent hematopoietic progenitor/stem cells (HPCs), develop in a specialized organ distinct from the bone marrow or the embryonic liver; *i.e.* the thymus (1). Thymic epithelial cells (TECs) are the specific components of the thymus microenvironment that provide unique inductive signals for keeping early thymic progenitors on track to T-cell differentiation (2–4). Two molecularly and functionally distinct TEC subsets are sequentially involved in T-cell development, cortical (c) TECs and medullary (m) TECs, which are located at the thymus cortex and medulla, respectively. cTECs impose T-cell commitment and induce the differentiation, expansion and positive selection of developing thymocytes, by providing continuous activation of the evolutionary conserved Notch signaling pathway (5–7) through the expression of the nonredundant Delta-like 4 (DLL4) Notch ligand (8, 9). Notch is a family of transmembrane receptors (Notch1 to Notch4 in mammals) with a major role in the regulation of critical processes such as cell fate specification, differentiation and proliferation/apoptosis in multiple cell lineages. Upon interaction with a membrane-bound specific ligand (Delta-like or Jagged in mammals), the intracellular domain of Notch (ICN) is proteolytically cleaved and released, entering the nucleus where it behaves as a transcriptional regulator of downstream genes, activating a particular genetic program (10, 11). In the thymus, progenitors that interact with TECs in the cortex activate the T-cell maturation program and then migrate to the medulla where mTECs promote their terminal differentiation and participate in central tolerance induction (12–14).

Despite its unique and crucial function in generating self-restricted and self-tolerant functional T cells throughout life, the thymus is the first organ to undergo aged-related involution. This is an evolutionary conserved process beginning as early as birth and no later than the onset of puberty in humans and mice (15). Thymic involution mainly results from the degeneration of the epithelial component of the thymic stroma and is characterized by dramatic reductions in thymus size and TEC numbers, the expansion of adipocytes and fibroblasts, and the disorganization of the thymic architecture, leading to diminished thymocyte numbers and reduced naïve T cell output (16–18). These features characterize as well the thymic involution process induced under physiological stress conditions such as infection, pregnancy, and cancer treatments (reviewed in 18). While several molecular mechanisms have been proposed to be involved in stress-induced acute thymic atrophy, the underlying mechanisms of chronic age-related involution remain less clear. Recent studies have documented many changes of TEC biology throughout life, revealing a surprisingly dynamic population with a high turnover (17). Therefore, understanding how TEC maintenance and regeneration are regulated in the adult thymus is of critical relevance for understanding thymic involution.

cTECs and mTECs arise early in ontogeny from a common thymic epithelial progenitor cell (TEPC) originated in the thymic primordium derived from the embryonic third pharyngeal pouch endoderm (19). This bipotent TEPC was identified in the fetal thymus (20–22) and its existence has been confirmed in the adult thymus (23–26), although the physiological contribution of bipotent TEPC to adult TEC generation remains controversial (27). In the embryo, differentiation of TEPCs into cTEC and mTEC lineages and development of a functional thymus is critically controlled by the transcriptional regulator Foxn1 (23, 28), which is induced in TEPCs by signals provided by other thymic components, including developing thymocytes (29–31). However, how cTEC/mTEC lineage specification and differentiation from the TEPC is induced has been a matter of intense debate. Studies showing that fetal TEPCs exhibit features and markers associated with the cTEC lineage (32, 33), support a serial progression model of TEC differentiation, in which cTEC lineage represents a default pathway, whereas mTEC specification from the common TEPC requires additional specific cues (34). The potential mechanisms controlling this mTEC specification step and the emergence of separate mTEC- and cTEC-restricted progenitors have remained poorly understood, although independent evidence has begun to emerge suggesting that the Notch pathway may be involved. In fact, signaling provided by the DLL1 Notch ligand induces maturation of fetal mTECs leading to the organization of medullary areas in a FTOC (35), while mice deficient in Jagged2 have thymi with reduced medullary areas (36). In the adult thymus, however, TEC-specific overexpression of active Notch leads to inhibition of mTEC lineage development and reduced TEC cellularity (37), indicating that Notch expression by TECs might be temporally regulated. Recently, two groups have provided genetic evidence that Notch signaling plays a crucial role at multiple embryonic stages during TEC development, but may be dispensable in postnatal life (38, 39). Importantly, they showed that Notch activation is required for maintenance/expansion of the undifferentiated TEPC and mTEC-restricted progenitor pools, and also for mTEC fate induction (38), while once the mTEC lineage was specified, further mTEC development was independent of Notch activity. Accordingly, repression of the Notch pathway was shown mandatory for progression of early mTECs to the mature mTEC stage (39), a fact that concurs with the downregulation of Notch activation in TECs after birth (35, 37). Collectively, these data have revealed a critical role of Notch as a potent regulator of TEPC homeostasis and mTEC lineage fate during fetal thymus development, although Notch function in the epithelial compartment of the postnatal thymus remains to be investigated. This is an important issue, regarding the hypothetical contribution of TEPC to adult TEC turnover (17, 23–27), which may impact the dynamics of thymus involution and its consequences to immunosenescence.

In this study, we have approached the potential contribution of Notch to postnatal TEC biology using two complementary strategies. First, we performed quantitative immunohistochemistry and confocal imaging approaches of *in situ* thymus Notch signaling (40) and provide evidence of a spatiotemporal regulation of *in vivo* Notch activation in both human and mouse postnatal TECs. Then, we made use of an *in vivo* genetic model of *Foxn1*-controlled conditional inactivation of Notch signaling in murine epithelial cells, including TECs, and reveal that lack of Notch signaling accelerates age-dependent loss of mTEC numbers and affects medulla integrity in the postnatal thymus. Therefore, we suggest a key role for Notch signaling in the control of postnatal mTEC homeostasis and age-dependent thymic involution.

## Materials and Methods

### Human and Mouse Thymus Samples

Human thymus biopsies were obtained from male and female Caucasian pediatric patients aged 3-days to 15-years undergoing corrective cardiac surgery, after informed consent was provided, and in accordance with the Declaration of Helsinki and to the procedures approved by the Spanish National Research Council Bioethics Committee.

Animal studies were reviewed and approved by the Animal Experimentation Ethics Committee of the Comunidad de Madrid, in accordance with the recommendations of the European Convention for the Protection of Vertebrate Animals Used for Experimental and Other Scientific Purposes (ETS 123). Mice were kept under specific pathogen-free conditions and used according to institutional regulations. C57BL/6J mice were obtained in-house from the departmental breeding facility. C57BL/6J *RBPjκ^fl/fl^* conditional knockout mice generated by Prof. Tasuku Honjo (41) and C56BL/6J *Rosa26^loxPLacZ^* reporter mice (Jackson Laboratory) were obtained from Dr. Jose Luis de la Pompa (CNIC, Madrid). The transgenic B6D2F1/J Foxn1-Cre line containing seven copies of the Cre transgene under the control of the *Foxn1* promoter has been previously generated (42), and heterozygous mutants (*Foxn1^Cre/+^*) were kept as a colony. Mice homozygous for a conditional deletion of RBPjκ specifically in epithelial cells were obtained by crossing *Foxn1^Cre/+^* heterozygous to *RBPjκ^fl/fl^* homocygous mice, followed by backcrossing of resultant *Foxn1^Cre/+^ RBPjκ^fl/+^* F1 heterozygous to *RBPjκ^fl/fl^* homocygous mice (**Supplementary Figure 1A**). Further selection of *Foxn1^Cre/+^x RBPjκ^fl/fl^* mice was performed by PCR genotyping (**Supplementary Material**). These mice, referred to as RBPjκ-KO^TEC^, displayed Cre-mediated *RBPjκ* deletion exclusively in epithelial cells, including TECs, but not in other thymic cells. Mouse gender was not considered in any experiment. *Foxn1^+/+^x RBPjk^fl/fl^* littermates were used as wild-type (WT) controls. Selection of crossed mice was performed by PCR genotyping of genomic DNA obtained by proteinase K (Sigma) digestion of 3 weeks-old mouse ear discs tissue, as described in **Supplementary Material**.

### Immunohistochemistry and Confocal Microscopy

Tissue samples were fixed in 4% paraformaldehyde/phosphate-buffered saline (PBS) [PFA/PBS, Sigma-Aldrich] and paraffin-embedded (Paraplast Plus, Sigma-Aldrich). Serial 8 μm sections were obtained from formalin-fixed paraffin-embedded (FFPE) slides that were mounted on poly-lysine-coated slides (SuperFrost UltraPlus, Thermo Fisher Scientific). Deparaffinised, rehydrated FFPE tissue slides were properly blocked as previously described (40). Tissue antigens were retrieved by boiling in sodium citrate (10 mM, pH 6.0) and endogenous peroxidase activity was quenched using 1% H2O2 100% methanol. For blocking of non-specific antibody binding sites, samples were incubated for 1h in blocking solution (3% bovine serum albumin, 20 mM MgCl2, 0.3% Tween 20, 5% fetal bovine serum in PBS), and permeabilized slides were incubated in blocking solution containing primary antibodies (**Supplementary Table 1**). Background and nonspecific staining was determined by incubating with Ig isotype-matched controls (**Supplementary Figures 2, 3**). Before addition of secondary antibodies, tissue endogenous biotin was quenched with Avidin/Biotin blocking solutions (Vector Laboratories). For Jag1 signal detection, tissue slides were incubated for 1 hour at RT with a horseradish peroxidase (HRP)-coupled anti-rabbit IgG secondary antibody (DAKO) and the signal was amplified using a Cyanine-3 Tyramide Signal Amplification (TSA-Cy3) Kit (NEL 744, 25 Perkin Elmer). For Notch1, Notch3, Notch4 and cleaved Notch1 (ICN1) signal detection, biotinylated anti-rabbit IgG secondary antibody (Vector Laboratories) was added before signal amplification with an Avidin/Biotin-HRP complex (Elite Vectastain ABComplex Kit, Vector Laboratories) and TSA-Cy3 Kit. For pan-cytokeratine (pCK) signal detection, Alexa Fluor dye-conjugated secondary antibodies were used (Thermo Fisher Scientific). The ABC-amplified signal was developed by adding Alexa Fluor 488- or Alexa Fluor 555-conjugated streptavidin (Thermo Fisher Scientific). Nuclei were stained with Topro3 (Thermo Fisher Scientific) and slides mounted with Fluoromount-G (SouthernBiotech).

Images were acquired using an LSM510 or an LSM900 laser scan confocal microscope (Zeiss) coupled to an Axio Imager.Z1 or an Axiovert 200 or an Axio Imager 2 (Zeiss) microscope using the following magnifications (Zeiss): 10× Plan-Neofluar (numeric aperture [NA] 0.3), 25× Plan-Neofluar [oil (NA 0.8)], 40×Plan-Neofluar [oil (NA 1.3)], 40xPlan-Apochromat [oil (NA 1.3)] and 63×Plan-Apochromat [oil (NA 1.4)]. Images were processed using ImageJ. Brightness and contrast were adjusted equally in samples and controls when needed. For defining nuclear (Hes1, ICN1 and Topro) regions of interest (ROIs), Otsu algorithm was used to select positive cells by intensity threshold (43) For defining pCK^+^ ROIs, Li algorithm (44) was used (**Supplementary Figure 3**). A median filter at 0.2 μm was used to remove noise before creating the selections.

Quantitative analyses of Hes1^+^ or ICN1^+^ cell numbers in thymus cortical or medullary regions was performed by using image thresholding (45). As TECs, and particularly cTECs, form an extensive network of finely branched cell processes, numbers of individual TECs in this network are difficult to define (46). Therefore, no quantitative measurements of TEC frequencies, especially of Hes1^-^/ICN1^-^ TECs, in the cortex *vs* the medulla could be performed. Rather, total numbers of Hes1^+^/ICN1^+^ nuclei within pCK^+^ ROIs were calculated relative to total Topro area or to pCK^+^ area defined in the thymus cortex or the medulla (**Supplementary Figure 3**). To this end, pCK^+^ ROIs were first defined as described above, and then used to create binary masks. Both nuclear (Hes1 or ICN1) and pCK binary masks where then processed on Image J’s “Image Calculator” using the logic operator “AND”. During image processing, a particular pixel intensity level (the threshold) is automatically defined by algorithms. Then, the number of pixels within the threshold is used to make a selection of ROIs, which exclusively contain the pCK-specific signal. The ROI is then used to calculate the total pCK^+^ area of TECs (in μm) and the number of Hes1^+^/ICN1^+^ nuclei within. Every cell out of the pCK ROI, including thymocytes positive for Notch activation markers, are systematically excluded and thus not considered in the analysis

Histomorphometric measurements of thymic cortex and medulla (**Supplementary Figure 4**) were also performed in ImageJ by ROIs using Jag1 and/or Topro intensity level threshold (43).

### Hematoxilin/Eosin and β Galactosidase (LacZ) Staining

Skin samples were fixed in 4% paraformaldehyde (PFA)/PBS solution (Sigma-Aldrich) and embedded in paraffin. Deparaffinised tissue slides were incubated for 3 min. in Harry’s hematoxilin (Sigma), washed and quickly differentiated (10 to 15 sec) in acid alcohol solution (0.5% HCl; 70% ethanol). Next, they were incubated for 9 min. in 0.5% (w/v) Eosin solution (Sigma) and sequentially dehydrated in graded ethanol series. Tissue slides were briefly incubated in xylene, mounted with Entellan mounting medium (Merck, Millipore), and analyzed with an optical microscope (DM2500; Leica) equipped with a CCD camera (DFC420; Leica), with Leica Application Suite software (version 4.3.0).

For β-galactosidase staining, thymic samples were fixed in 0.125% glutaraldehyde/PBS solution, washed (0.02% Nonidet-P40, 0.11% sodium deoxycholate, and MgCl2 2mM in phosphate buffer 0.1M, pH 7.3) and stained with X-gal staining solution (washing buffer supplemented with potassium ferricyanide 5mM, potassium ferrocyanide 5mM and 1mg/ml of X-gal resuspended in N,N-dimethyformamide). Samples were then washed, fixed in 4% PFA/PBS and paraffin-embed. Sections (8 μm) were mounted on poly-lysine-coated slides (SuperFrost Ultra Plus, Thermo Scientific) and deparaffinised as specified earlier. Cell nuclei were stained with Nuclear Fast Red (Vector Labs), sequentially dehydrated in graded ethanol series and xylene, and mounted with Entellan mounting medium (Merck, Millipore).

### Flow Cytometry

For flow cytometry TEC analysis, thymus samples from either RBPjκ-KO^TEC^ or *Foxn1^+/+^x RBPjk^fl/fl^* control littermates, no separated by gender and aged from 0.5- to 12-months, were dissociated in RPMI medium (1.25 mg/ml collagenase D (Roche) following three digestion steps of 15 min at 37° C. Isolated cells were then diluted in RPMI1640 medium with 10% FBS (Gibco) containing DNaseI (Roche; 0.05 mg/ml). After filtering cell suspension through 70 μm cell strainer (Filcon) to remove clumps, flow cytometry was performed using a sequential gating strategy (**Supplementary Figure 5**) on cells stained with DAPI (Beckman Coulter) to exclude dead cells, anti-CD45-FITC (eBioscience) and anti-TER-119-FITC (Biolegend) mAb, to exclude hematopoietic and erythroid-lineage cells. Anti-MHCII-PECy7 (eBioscience) and anti-EpCAM-APCCy7 (Biolegend) was used to electronically gate TECs. EpCAM-gated TEC cells were then analyzed for reactivity with the anti-Ly51-PE (eBioscience), and UEA-1 biotinilated (Vector Labs) cTEC and mTEC-specific mAbs, respectively, developed using Streptavidin-APC (Biolegend).

For thymocyte flow cytometry, Ficoll-Hypaque (Lymphoprep, Axis-Shield PoC AS)-separated thymus cell suspensions were stained with the following mAbs: anti-CD8-FITC (Life Technologies), anti-CD4-PE (BD Biosciences), anti-CD19-PE (eBioscience), anti-CD90 (Thy1) (Biolegend), anti-CD11b-FITC (BD Biosciences), anti-B220-PE-Cy5 (Life Technologies), anti-NK1-APC (BD Biosciences). Anti-Ly5.1 and anti-Ly5.2 mAbs (BioLegend) were used in adoptive transfer experiments. Acquisition and analysis was performed in a FACSCanto II (BD Biosciences). All flow cytometry data were analyzed using FlowJo Version 10.0.7.2.

### Adoptive Cell Transfer

For the generation of BM chimeric mice, cell suspensions were isolated by Fycoll-Hypaque from BM samples obtained from femurs of 9 weeks-old RBPjκ-KO^TEC^ (Ly5.2^+^) mice, and BM cells (5x10^6^) were resuspended in 100μl of sterile PBS and injected i.v. into 8 weeks-old C57BL/6 (Ly5.1^+^) hosts (n=4) subjected to lethal irradiation (10 Gys) the day before. Recipient mice were euthanized 4 months post-transplantation and thymus reconstitution by Ly5.2^+^ cells was analyzed by flow cytometry. As control, BM cells from *Foxn1^+/+^x RBPjk^fl/fl^* WT littermates (Ly5.2^+^) were injected into C57BL/6 (Ly5.1^+^) irradiated hosts (n=2).

### Statistics

Statistical analysis was performed with GraphPad Prism 7.0 Software. The normal distribution of the data was tested using the Shapiro–Wilk normality test. When comparing two means of normal data, statistical significance (p) was determined by the unpaired two-tailed Student’s t-test. When comparing two-means of non-normal data, statistical significance (p) was determined by the unpaired Mann-Whitney test. When comparing more than two groups of normal data, one-way ANOVA was used, and for no normal data Klustal-Wallis was used. When comparing groups of two independent variables, two-way ANOVA was used. In all cases, the a-level was set at 0.05. Data in graphs are presented as mean ± SEM.

## Results

### Notch Signaling Is Active *In Vivo* in Human and Mouse Postnatal TECs

**Figure 1:**
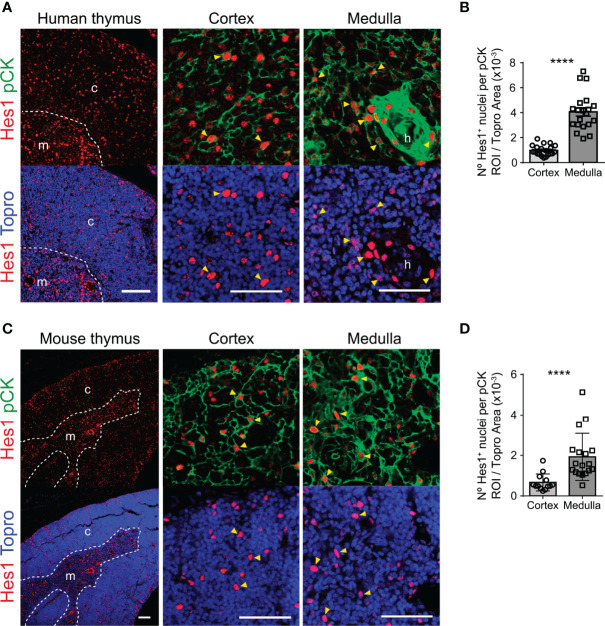
Notch signaling is active *in vivo* in human and mouse postnatal TECs. Immunohistochemistry of the canonical Notch target Hes1 (red) in postnatal human (≤ 6-years) and mouse (≤ 5-months) thymus. TECs are characterized by expression of pCK (green). Topro3 shows nuclear staining (blue). **(A)** General view (scale bar: 100μm) of Hes1 expression in a representative human thymus sample (18-months), and detailed view (scale bar: 50μm) of Hes1 and pCK expression in the thymus cortex and medulla. Dotted line, corticomedullary junction; c, cortex; m, medulla. Arrowheads indicate Hes1 expression in TECs (pCK^+^); h, Hassal’s corspuscles. **(B)** Bar graphs show numbers of Hes1^+^ nuclei within pCK^+^ ROIs relative to total (Topro^+^) cellular areas analyzed in the human thymus cortex and medulla. Data are shown as mean numbers ± SEM per field obtained from n= 8-10 different 63x images from sample, (n ≥ 2 independent human thymus samples aged ≤ 6-years). ****p < 0.0001. **(C)** General view (scale bar: 100μm) of Hes1 expression in a representative mouse thymus sample (5 months), and detailed view (scale bar: 50μm) of Hes1 and pCK expression in the thymus cortex and medulla. Dotted line, corticomedullary junction; c, cortex; m, medulla. Arrowheads indicate Hes1 expression in TECs (pCK^+^). **(D)** Bar graphs show numbers of Hes1^+^ nuclei within pCK^+^ ROIs relative to total (Topro^+^) cellular areas analyzed in the mouse thymus cortex and medulla. Data are shown as mean numbers ± SEM per field obtained from n= 10 different 63x images from sample, (n ≥ 2 independent mouse thymus samples aged ≤ 5-months). ****p < 0.0001.

Detailed analyses of Notch activation in postnatal TECs are scarce in mice and remain to be performed in humans. We approached this issue by three-color immunohistochemistry and quantitative confocal microscopy of several postnatal human (≤ 6-years) and mouse (≤ 5-months) thymus samples labelled with a mAb recognizing the well-established target of canonical Notch signaling Hes1 (13), together with a TEC-specific anti-pCK mAb mix, and with Topro3 for nuclear staining. General examination of representative thymus sections stained with anti-Hes1 and Topro3 revealed a continuous pattern of nuclear Hes1 expression throughout the whole human thymus, which seemed more prominent at the medulla and was similar in the mouse postnatal thymus. Detailed analyses aided by the co-staining with anti-pCK, allowed the identification of Hes1^+^ pCK^+^ TECs at the thymus cortex and medulla in both species (**Figures 1A, C**). Hes1^+^ cells lacking the pCK TEC maker, characterized in previous studies as developing thymocytes (40), were also identified distributed throughout the inner cortex in both human and mouse thymi; while, as shown previously (40), Hes1^+^ thymocytes seemed less abundant at the medulla, suggesting that Hes1 expression at the medulla occurs mostly in TECs. (**Figures 1A, C**). A significant fraction of such Hes1^+^ mTECs, which displayed the highest Hes1 expression levels, was found accumulated in Hassal’s corpuscles (HCs) in the human thymus (**Figure 1A**). Therefore, these results indicate that Notch activation is conserved in postnatal cTECs and mTECs. Quantitative confocal analyses based on thresholding image approaches (45; **Supplementary Figure 3**), confirmed that measurable numbers of Hes1^+^ nuclei were distributed within the cortical and medullary pCK^+^ areas analyzed in both human and mouse postnatal thymus samples, with Hes1^+^ cells being more abundant at the medulla in both species (**Figures 1B, D**). However, no frequencies of Hes1^+^ cTECs *versus* mTECs could be establish by this approach, as TECs, and particularly cTECs, have a complex morphology and display a high intrathymic cellular density (46), making it difficult to identify individual TECs and to define TEC numbers within particular ROIs. Collectively, these analyses provide the first direct evidence that Notch signaling is active *in vivo* in the human postnatal thymus, in TECs located both at the cortex and the medulla, and confirm that activation of Notch is also induced after birth in the mouse thymus, pointing to a conserved role for Notch signaling in postnatal TEC biology.

### The Notch1 Receptor Mediates *In Vivo* Activation of Notch Signaling in Human Postnatal mTECs

**Figure 2:**
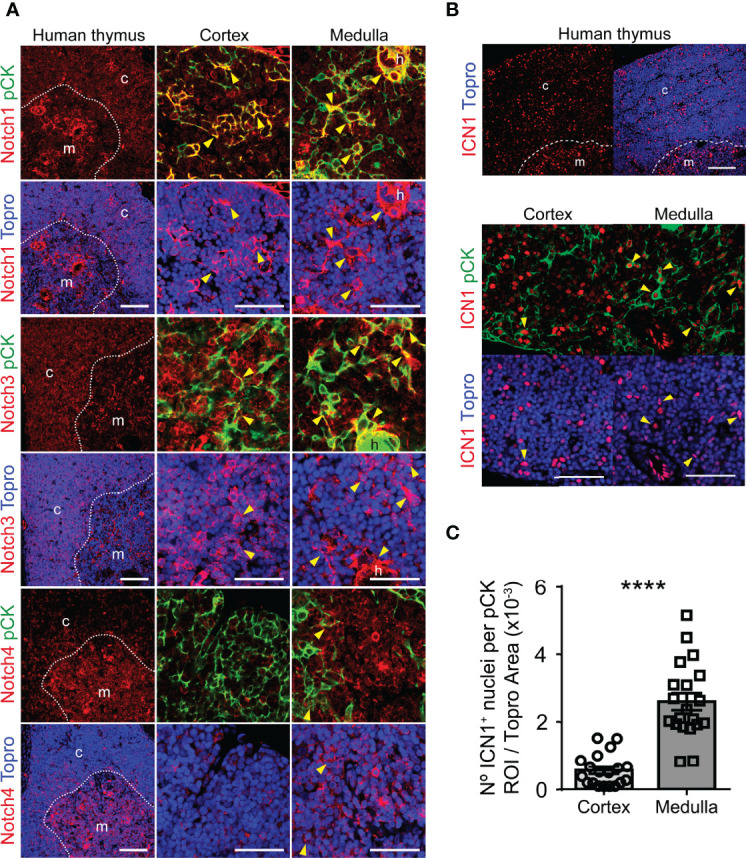
Expression of Notch receptors and activation of Notch1 signaling in human postnatal thymus. **(A)** Immunohistochemistry of the indicated Notch receptors in the human postnatal thymus (≤ 18 months). TECs are characterized by pCK expression (green). Topro3 shows nuclear staining (blue). General view (scale bar: 100μm) of Notch receptor distribution in representative human postnatal thymus samples, and detailed view (scale bar: 50μm) of Notch receptor expression by TECs located at the cortex and medulla. Dotted line, corticomedullary junction; c, cortex; m, medulla. Arrowheads indicate Notch receptor expression by TECs (pCK^+^). Images shown are representative of n ≥ 5 different 63x images from n = 2 independent human thymus samples. **(B)** Immunohistochemistry of active intracellular Notch1 (ICN1) (red) in representative human postnatal thymus samples (≤ 6-years). TECs are characterized by expression of pCK (green). Topro3 shows nuclear staining (blue). General view (scale bars: 100μm) of ICN1 expression (top) and detailed view (scale bars: 50μm) of ICN1 and pCK staining in human thymus cortex and medulla (bottom). Dotted line, corticomedullary junction; c, cortex; m, medulla. Arrowheads indicate ICN1 expression in TECs. **(C)** Bar graphs showing numbers of ICN1^+^ nuclei within pCK^+^ ROIs relative to total (Topro^+^) cellular areas analyzed in human thymus cortex and medulla samples. Data are shown as mean numbers ± SEM per field obtained from n=10 different 63x images per thymus sample (n=3 independent thymus samples aged ≤ 6-years), ****p < 0.0001.

While murine fetal TECs express several Notch receptors (35, 38, 39), genetic evidence has been provided that Notch1 is the receptor responsible for Notch activation in mouse embryonic TECs (39). To begin to decipher which Notch receptor/s is responsible for *in vivo* Notch signaling in the human thymus, we analyzed *in situ* Notch receptor expression in tissue sections of human postnatal thymus labeled with the anti-pCK mAb in combination with a mAb specific for either Notch1, Notch3 or Notch4. Immunohistochemistry and confocal microscopy showed that, as expected from previous studies (40), Notch1 is broadly expressed by pCK-negative thymocytes distributed mostly throughout the cortex. In addition, Notch1 was expressed by a minor population of pCK^+^ cTECs and by a significant number of mTECs (**Figure 2A**). Notch3 displayed an expression pattern similar to Notch1, and was significantly expressed by cortical thymocytes, but only by few cTECs, while substantial numbers of mTECs coexpressed pCK and Notch3 (**Figure 2A**). In contrast, Notch4 expression was essentially confined to a non-epithelial pCK^-^ population located at medulla, which has previously been characterized as dendritic cells (47), although rare Notch4^+^ mTECs could be identified as well (**Figure 2A**). Therefore, as shown before for mouse fetal TECs (39), Notch1 may be the preferential receptor that mediates Notch signaling *in vivo* in human postnatal TECs in both cortex and medulla, with a possible contribution of Notch3 in mTECs.

To directly investigate the contribution of Notch1 to *in vivo* activation of Notch signaling in human postnatal TECs, we performed immunohistochemistry and confocal microscopy, using a mAb against the active intracellular form of Notch1 (ICN1) in combination with anti-pCK antibodies. These analyses confirmed Notch1 activation *in situ* in the human postnatal thymus, revealing nuclear expression of ICN1 in cells distributed throughout both the cortex and the medulla (**Figure 2B**). As shown before (40), we found that significant numbers of cells expressing active Notch1 in the cortex were pCK-negative hematopoieitic cells, although ICN1^+^ cTECs were also identified, while cells that display Notch signaling at the medulla seemed to be mostly pCK^+^ mTECs (**Figure 2B**). Quantitative analyses of imaging data allowed to measure significant numbers of ICN1^+^ nuclei within the pCK^+^ cortical and medullary areas (**Figure 2C**), supporting that both cTECs and mTECs activate Notch1 *in vivo*. Collectively, the observed ICN1 expression pattern suggests that the Notch1 receptor contributes significantly to *in vivo* activation of Notch signaling in human postnatal TECs.

### Activation of Notch Signaling Increases With Thymus Age in Postnatal mTECs

**Figure 3:**
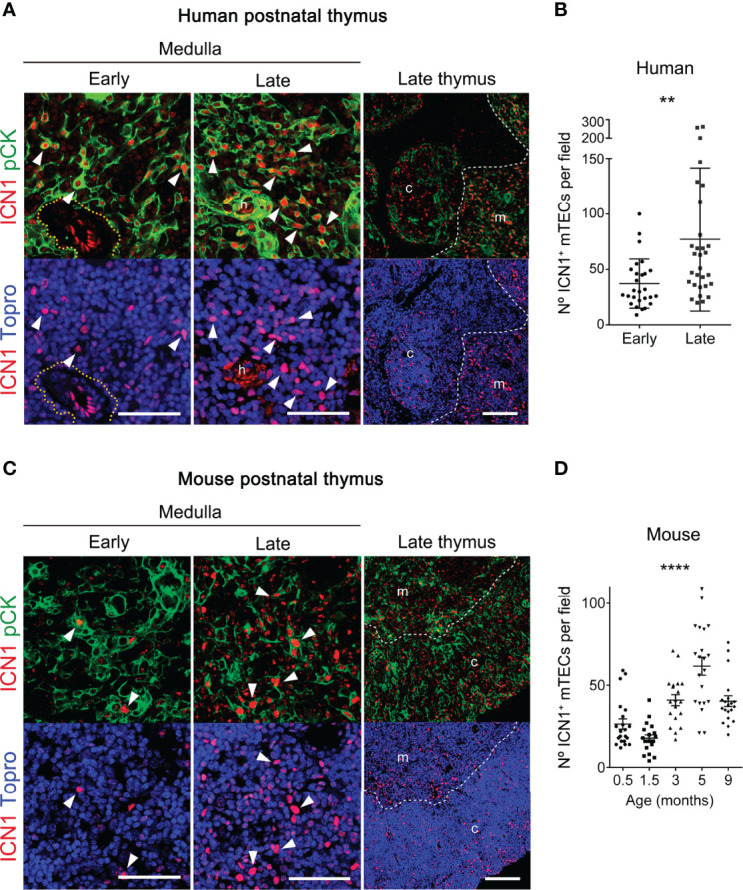
Notch1 signaling increases with thymus age in postnatal mTECs. **(A, C)** Immunohistochemistry of active intracellular NOTCH1 (ICN1, red) and pCK (green), with nuclei in blue (Topro3) either in human thymus samples representative of early (≤ 1.5-years) and late (6-13 years) postnatal ages **(A)**, or in mouse early (≤ 4-weeks) and late (3-9 months) postnatal thymi **(C)**. General views (scale bar: 100μm) of ICN1 and pCK expression in representative late thymus samples are shown on the right. Detailed views (scale bars: 50μm) of ICN1 and pCK expression at the early and late thymus medulla are shown in the left and middle panels, respectively. Dotted line defines the perivascular space; * indicates endothelial cells expressing ICN1; h, Hassal’s corspuscles. Arrowheads indicate ICN1 expression in pCK^+^ TECs. Images are representative of n ≥ 10 different images from independent sample (n ≥ 3 thymus samples). **(B)** Numbers of ICN1^+^ pCK^+^ mTECs in human thymus samples representative of early (≤1.5-years) and late (6-13 years) postnatal ages labelled as in **(A)**. Data are shown as mean numbers ± SEM per field obtained by counting ICN1^+^ pCK^+^ medullary cells from n ≥ 10 different 63x images per thymus sample (n= 3 independent thymus samples from each group of age), **p < 0.01. **(D)** Numbers of ICN1^+^ pCK^+^ mTECs in thymus samples of mice aged from 0.5- to 9-weeks, labelled as in B). Data are shown as mean numbers ± SEM per field obtained by counting ICN1^+^ pCK^+^ medullary cells from n=10 different 63x images per thymus sample (n= 2-3 independent thymus samples from each group of age), ****p < 0.0001.

In the course of our studies on *in vivo* activation of Notch signaling, we noticed a consistent heterogeneity of ICN1^+^ cell numbers among human thymus samples at distinct postnatal ages from 1-month to 6-years. Considering that significant physiological changes occur in the human thymus during the first few years of life (15), we wanted to investigate the possibility that activation of Notch signaling could be regulated along time in the postnatal thymus. To this end, we performed quantitative immunohystochemistry and confocal microscopy of ICN1 expression in two groups of human thymus samples representative of early (≤1.5 years) and late (6-13 years) postnatal ages. The selected groups were expected to differ in age-dependent physiological features associated to thymic involution, as regression of the thymic epithelium can be observed early in life in humans, long before puberty (reviewed in 15). As current data in mice have shown that *in vivo* Notch activation during thymopoiesis is selectively induced in medullary-lineage TECs (38, 39), age-dependent Notch activation was specifically analyzed in the thymus medulla. We thus performed detailed image analyses of ICN1 and pCK expression in mTECs and found that activation of Notch1 signaling was more prominent in the medulla of late compared to early human postnatal thymi (**Figure 3A**). Although morphologically heterogeneous, mTECs are less dense than cTECs, and therefore more easily defined as individual cells (46), allowing us to perform quantitative measurements of pCK^+^ cells expressing nuclear ICN1, as shown in **Supplementary Figure 3B**. These analyses revealed that numbers of mTECs expressing ICN1 increased 50% on average in the late compared to the early human postnatal thymus (**Figure 3B**), supporting an age-dependent activation of Notch1 signaling in mTECs. Then, we investigated whether this progressive increase of mTECs expressing active Notch1 could be observed in mice. To this end, we performed quantitative analyses of *in situ* Notch1 activation in mTECs from mice aged 2-weeks to 9-months (**Figures 3C, D**). The results showed a slight, but not significant, decrease in the numbers of murine mTECs that expressed active Notch1 during the first weeks of life from 0.5 to 1.5 months of age (**Figure 3D**), coincident with the period of neonatal thymus growth (17). However, ICN1^+^ mTEC numbers increased significantly by 3 months, and up to 4-fold by 5 months (**Figure 3D**), confirming a highly significant age-dependent upregulation of Notch1 activation in postnatal mouse mTECs. Collectively, the observed age-associated activation of Notch1 signaling in the postnatal thymus of both humans and mice suggests a conserved role for Notch1 signaling in the biology of postnatal mTECs.

### *Foxn1*-Controlled *RBPjκ* Deletion Abrogates Canonical Notch Activation in Postnatal TECs

To better understand the contribution of the Notch pathway to postnatal mTEC biology, we next analyzed the impact of impaired Notch activation in TECs, by using a conditional loss-of-function mouse model, in which canonical Notch signaling was selectively abolished in epithelial cells by crossing Foxn1-Cre mice (42) to the *Rbpj^fl/fl^* conditional knockout mouse line (41) (**Supplementary Figure 1A**). Transgenic Cre expression in Foxn1-Cre mice parallels endogenous *Foxn1* expression in epithelial cells and can be detected as early as E10.5 in the thymus primordium (42). Crossing Foxn1-Cre mice to the Rosa26^loxPlacZ^ reporter strain has revealed Foxn1 protein expression at E11.5, while *Foxn1*-controlled β-galactosidase reporter expression detected by LacZ staining is induced at E12.5 (42), and can be observed in the postnatal thymus as well (**Supplementary Figure 1B**). Therefore, RBPjκ in Foxn1-Cre x *Rbpj^fl/fl^* homozygous mice (hereafter referred to as RBPjκ-KO^TEC^) might not be abolished before E11.5-12.5, which corresponds to a time in development when TEC progenitors have been established and their progeny has contributed to an initial thymus primordium. At later stages, emerging TECs and skin epithelial cells (42), will be unable to activate the canonical Notch signaling pathway in mutant mice (41). Confirming Notch abrogation in skin epithelial cells, RBPjκ-KO^TEC^ mice developed macroscopic cutaneous lesions, which were evident at 8-months, when animals showed clear signs of disease including numerous lesions at the face, footpad, tail and ventral skin (**Supplementary Figure 1C**). Microscopic examination of these lesions revealed a clear disorganization of the skin with signs of inflammation, leukocyte infiltration, hair follicle hyperproliferation, and the generation of keratin cysts (**Supplementary Figure 1D**), consistent with previous observations in distinct mouse models of Notch-deficient skin epithelium (48, 49).

**Figure 4:**
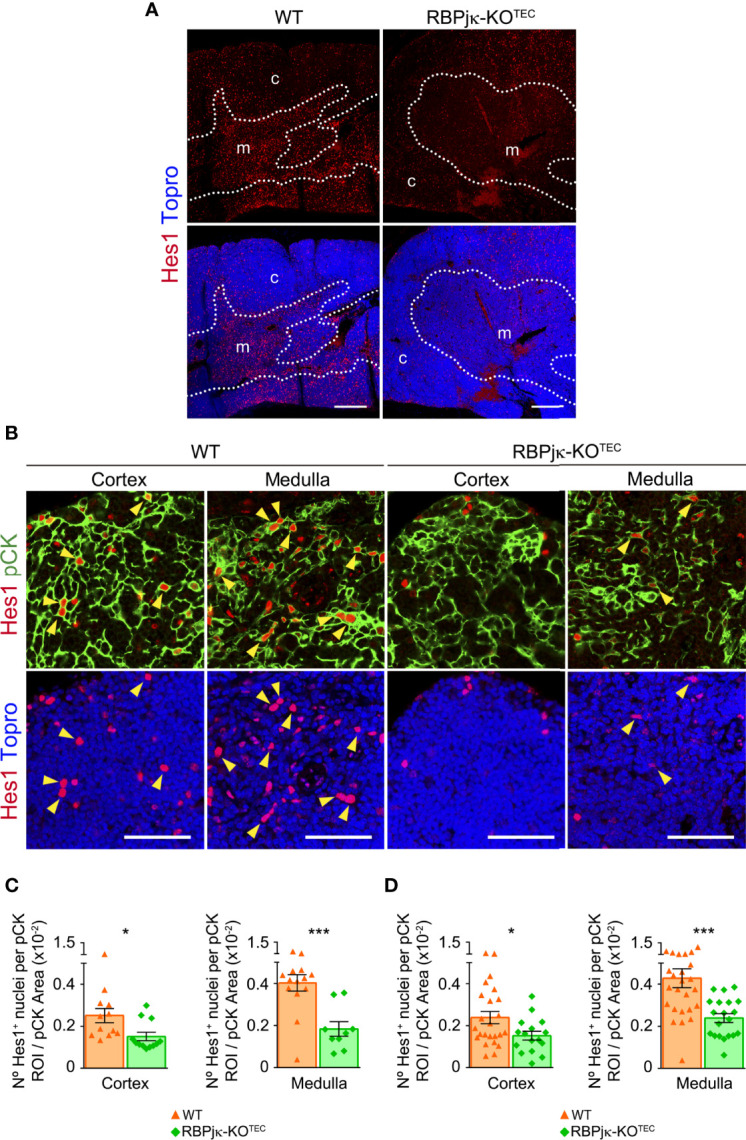
Abrogation of canonical Notch signaling in postnatal TECs of RBPjκ–KO^TEC^ mice. **(A)** General view of Hes1 expression (red) in postnatal thymi (5-months) of mutant RBPjκ–KO^TEC^ and WT *Foxn1^+/+^x RBPjk^fl/fl^* littermate mice. Topro3 shows nuclear staining (blue). Scale bar: 200μm. c, cortex; m, medulla. Dotted line, corticomedullary junction. **(B)** Immunohistochemistry of Hes1 (red), and pCK (green) with nuclei in blue (Topro3) in the cortex and medulla of postnatal thymi (5-months) from WT and RBPjκ–KO^TEC^ mice. Arrowheads indicate Hes1 nuclear expression. Scale bars: 50μm. Images are representative of n ≥ 10 images per sample (n ≥ 3 independent thymus samples). **(C, D)** Bar graphs show numbers of Hes1^+^ nuclei within pCK^+^ ROIs relative to total pCK^+^ cellular areas analyzed in the thymus cortex and medulla of mutant RBPjκ–KO^TEC^ and WT *Foxn1^+/+^x RBPjk^fl/fl^* littermate mice aged 3-months **(C)** or 5-9-months **(D)**. Data are shown as mean numbers ± SEM per field obtained from n ≥ 10 different 63x images per sample (n=4 independent samples). *p < 0.05; ***p < 0.00.

Having confirmed the loss of Notch activation in the skin of RBPjk-KO^TEC^ mice, we next investigated specific abrogation of Notch signaling in mutant postnatal TECs (≥ 5-months), as compared to *Foxn1^+/+^x RBPjk^fl/fl^* WT littermate controls. To this end, we performed comparative immunohistochemistry of Hes1 expression as readout of canonical Notch activation. Consistent results showed a prominent expression of Hes1 in the medulla of WT postnatal thymi, which was drastically reduced in RBPjk-KO^TEC^ mutant thymi, confirming abrogation of Notch signaling (**Figure 4A**). Detailed examination of the cortical and medullary TEC niches (**Figure 4B**) confirmed that, as shown above (**Figures 1**, **3**), TECs that display Notch signaling *in vivo* represent a conspicuous population in the medulla of WT thymi, and Hes1^+^ TECs were also detected in the WT cortex (**Figures 4C, D**). Quantitative measurements of Hes1^+^ nuclei distributed within pCK^+^ ROIs (**Supplementary Figure 3**) revealed a significant reduction of nuclei expressing Hes1 in both the cortex and the medulla of mutant RBPjκ-KO^TEC^ thymi compared to WT thymi of mice aged 3-months (**Figure 4C**), and a similar reduction was maintained in mice of 5-9-months (**Figure 4D**), which was consistently more significant in the medulla than in the cortex (**Figures 4C, D**). Collectively, these results confirmed that *Foxn1*-controlled abrogation of RBPjk impairs canonical activation of the Notch pathway in a substantial population of mTECs and also in a subset of cTECs in the postnatal thymus of RBPjκ-KO^TEC^ mutant mice.

### *Foxn1*-Controlled Abrogation of Notch Signaling Leads to an Accelerated Loss of Postnatal mTECs

**Figure 5:**
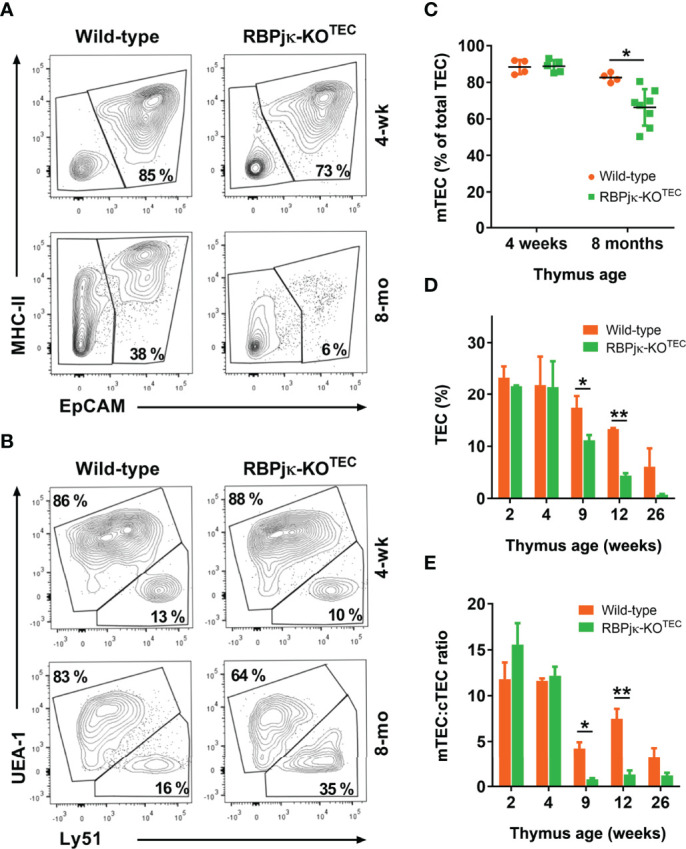
*Foxn1*-controlled abrogation of canonical Notch signaling leads to an accelerated age-associated loss of postnatal mTECs. **(A)** Representative flow cytometry MHC-classII and EpCAM expression analysis of CD45- and Ter-119-depleted cell suspensions of collagenase/dispase-treated young (4-weeks) and old (8-months) thymi from RBPjκ–KO^TEC^ mutant and *Foxn1^+/+^x RBPjk^fl/fl^* littermate WT mice (n ≥ 8). **(B)** Representative flow cytometry analysis of UEA1 and Ly51 expression on gated EpCAM+ TECs in **(A)**, (n ≥ 4). **(C)** Relative numbers of UEA1^+^ mTECs among EpCAM^+^ TECs from RBPjκ–KO^TEC^ and *Foxn1^+/+^x RBPjk^fl/fl^* WT thymi of the indicated ages, analyzed as in B). Data are shown as mean percentages ± SEM (n ≥ 4 thymus samples per age). **(D)** Relative numbers of EpCAM^+^ TECs present in cell suspensions obtained as in **(A)** from thymi of *Foxn1^+/+^x RBPjk^fl/fl^* WT and RBPjκ–KO^TEC^ mutant mice aged 2- to 26-weeks. Data are shown as mean percentages ± SEM (n ≥ 3 thymus samples per age). **(E)** Ratio of mTEC:cTEC proportions among total EpCAM^+^ TECs from WT *Foxn1^+/+^x RBPjk^fl/fl^* and mutant RBPjκ–KO^TEC^ thymi at the indicated postnatal ages. Data show mean values ± SEM (n ≥ 4 thymus samples per age). *p<0.05; **p<0.01. p values were calculated using a two-tailed t-test.

To investigate the impact of the specific abrogation of Notch signaling in the TEC compartment of the postnatal thymus, we next performed flow cytometry to analyze the TEC composition of thymi isolated from mutant RBPjκ-KO^TEC^ mice and *Foxn1^+/+^x RBPjk^fl/fl^* WT littermates at different postnatal ages. To this end, cell suspensions from collagenase-dissociated thymi were analyzed for expression of EpCAM and MHC-class II (MHC-II) TEC markers after electronic exclusion of hematopoietic and erythroid-lineage cells by gating off CD45^+^ and Ter119^+^ cells (**Supplementary Figure 5**). Absolute and relative cell counts of EpCAM^+^ cells revealed no significant numerical differences of total TECs between RBPjκ-KO^TEC^ and WT thymi at early (4-weeks) postnatal ages, while TEC proportions decreased significantly in late (8-months) postnatal thymi of RBPjκ-KO^TEC^ mutant mice (**Figure 5A**). As we found that active Notch is expressed *in vivo* in mTECs in an age-dependent manner, we assessed whether the observed decrease of TEC numbers in aged mutant mice was the result of a preferential loss of mTECs. Thus, we then quantified cTECs and mTECs among EpCAM^+^ TECs by FACS analyses based on expression of the specific Ly51 and UEA1 markers, respectively (**Figure 5B**). No significant differences were observed in the proportions of either cTECs or mTECs in RBPjκ-KO^TEC^ compared to WT thymi at 4-weeks of age, while relative mTEC numbers decreased significantly in thymi from 8-months-old RBPjκ-KO^TEC^ mice compared to *Foxn1^+/+^x RBPjk^fl/fl^* control littermates (**Figures 5B, C**). Therefore, TEC-specific loss of Notch signaling results in a marked decrease in the proportions of TECs in late but not early RBPjκ-KO^TEC^ mutant thymi, which results in a preferential reduction of mTECs. To further assess the kinetics of mTECs loss, we performed quantitative flow cytometry analyses of cTEC and mTEC numbers in mutant and WT littermates aged from 2- to 26-weeks. We found no significant differences in relative TEC numbers between the two groups at young postnatal ages (2- and 4-weeks) However, TEC proportions decreased markedly at 9-weeks in RBPjκ-KO^TEC^ compared to WT littermate mice, and this decrease progressed steadily to 26-weeks (**Figure 5D**). Importantly, we found that WT mice also displayed a progressive age-dependent decrease of relative TEC numbers, as previously reported (17), although loss of TECs in mutant mice followed accelerated kinetics compared to WT littermates (**Figure 5D**). Therefore, impaired Notch signaling in TECs results in a marked acceleration of TEC number loss in the postnatal thymus. Independent quantification of relative cTEC and mTEC numbers revealed a preferential decrease of mTECs along age in both WT and mutant mice, which led to a significant reduction of the mTEC:cTEC ratio in both mouse groups by 9-weeks (**Figure 5E**). The mTEC:cTEC ratio was maintained to minimal levels up to 26-weeks in RBPjκ-KO^TEC^ thymi, and the decrease was less pronounced in the thymus of WT littermates (**Figure 5E**). Therefore, our results indicate that in both RBPjκ-KO^TEC^ and WT mice, the observed age-associated decrease of postnatal TEC numbers can be attributed to a preferential loss of mTECs. However, abrogation of Notch signaling in RBPjκ-KO^TEC^ mutant mice leads to an accelerated loss of postnatal mTECs, suggesting that Notch activation regulates mTEC homeostasis in postnatal life.

### Abrogation of Canonical Notch Signaling in TECs Leads to a Reduced and Disorganized Postnatal Thymic Medulla and Accelerates Thymic Involution

**Figure 6:**
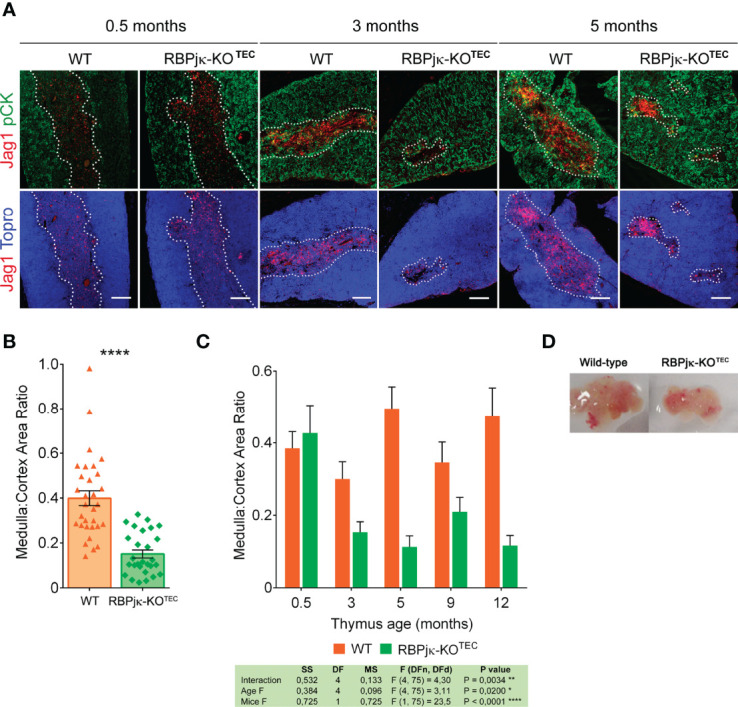
Abrogation of Notch signaling in TECs results in thymic medulla disruption and thymus atrophy. **(A)** Immunohistochemistry of thymi from mutant RBPjκ–KO^TEC^ and WT *Foxn1^+/+^x RBPjk^fl/fl^* littermate mice at 0.5-, 3- and 5-months of age. TECs are characterized by expression of pCK (green), Jag1 Notch ligand (red) expression marks medullary TECs, and Topro3 shows nuclear staining (blue). Images are representative of n ≥ 10 images per sample (n ≥ 3 thymus samples per age). Scale bar: 200μm. **(B)** Ratio of medulla: cortex area measurements derived from histomorphometric analysis (**Supplementary Figure 4**) of postnatal thymi from WT *Foxn1^+/+^x RBPjk^fl/fl^* and mutant RBPjκ–KO^TEC^ mice in A). Coexpression of pCK and Jag1 (confined to the medulla) was used to calculate medullary areas. Cortical areas were identified as pCK^+^ Jag1^-^ and nuclear staining by Topro3 (blue) defined total thymic area. Data are shown as mean area ratios ± SEM obtained from n ≥ 10 images per sample (n ≥ 3 thymus samples per age). p values were calculated using a two-tailed t-test. **(C)** Ratio of medulla: cortex area measurements derived from histomorphometric analyses as in **(B)** of thymi obtained from WT *Foxn1^+/+^x RBPjk^fl/fl^* and mutant RBPjκ–KO^TEC^ mice at the indicated ages. Data are shown as mean area ratios ± SEM obtained from n ≥ 10 images per sample (n ≥ 3 thymus samples per age). Two-way ANOVA table summarizing the statistical analysis is shown *p<0.05; **p<0.01. **(D)** Thymus atrophy in RBPjκ–KO^TEC^ mice mutant mice at 3-months of age.

Age-dependent mTEC loss occurs in normal thymus as part of the thymic involution process (17). It is thus possible that Notch signaling may contribute to the control of mTEC homeostasis and age-dependent thymus involution in postnatal life. As thymic regression results in loss of thymic structure and disorganization of thymic architecture (reviewed in 18), we next performed histomorphometric analyses aimed at establishing detailed comparisons between the cortical and medullary compartments of postnatal thymi from RBPjκ-KO^TEC^ mice and *Foxn1^+/+^x RBPjk^fl/fl^* WT littermates. Expression of the Notch ligand Jag1, which is selectively expressed on TECs located at the medulla (40), was used to define the medullary microenvironment (**Supplementary Figure 4A**). Cortical and medullary area measurements by confocal microscopy revealed no significant differences in size and morphology of the cortex and medulla of young (0.5-months) thymi from RBPjκ-KO^TEC^ mice, as compared with WT littermates. However, a significant reduction of the medullary area was evident at 3 and 5 months of age in RBPjκ-KO^TEC^ thymi (**Figure 6A**). Compared to the WT thymic medulla, the mutant medulla appeared disorganized and composed of small discrete islets (**Figure 6A**), suggesting that TEC-specific abrogation of Notch signaling leads to the disruption of the medullary thymic microenvironment. Accordingly, histomorphometric measurements of cortical and medullary areas revealed a significant decrease of the average medulla to cortex area ratio of RBPjκ-KO^TEC^ thymi compared to WT thymi from the 3- and 5-months-old mice analyzed (**Figure 6B**). Importantly, kinetic studies based on histomorphometric measurements of postnatal thymi at increasing ages, from 0.5- to 12-months, revealed that the significant reduction of the medulla to cortex area ratio observed in RBPjκ-KO^TEC^ thymi at 3-months of age was progressive along life (**Figure 6C**). Also, macroscopic examination revealed that the observed medulla reduction correlated with postnatal thymus atrophy in mutant mice that was evident by 3-months (**Figure 6D**). Collectively, these data indicate that maintenance of the anatomical organization and integrity of the postnatal thymic medulla critically depends on the activation of the canonical Notch signaling pathway in mTECs.

**Figure 7:**
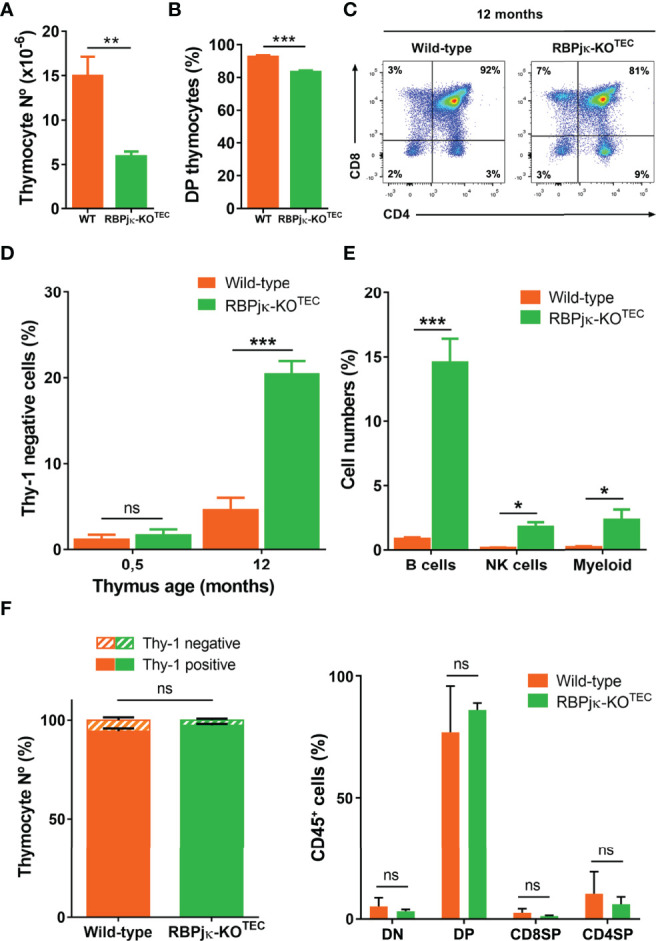
Abrogation of canonical Notch activation in TECs results in premature thymic dysfunction. **(A)** Absolute numbers of total thymocytes isolated from 12-month-old WT *Foxn1^+/+^x RBPjk^fl/fl^* or mutant RBPjκ–KO^TEC^ thymi. Data show mean numbers ± SEM, (n ≥ 4). **(B)** Percentages of CD4^+^CD8^+^ DP thymocytes among Thy1^+^ thymic cells from 12-month-old WT *Foxn1^+/+^x RBPjk^fl/fl^* or RBPjκ–KO^TEC^ mice. Data show mean percentages ± SEM, (n ≥ 4). **(C)** Representative flow cytometry analysis of CD4 and CD8 expression on gated Thy1^+^ thymocytes from 12-months-old WT *Foxn1^+/+^x RBPjk^fl/fl^* and RBPjκ–KO^TEC^ thymi, (n ≥ 4). **(D)** Percentages of thymic cells lacking Thy1 obtained from WT *Foxn1^+/+^x RBPjk^fl/fl^* or RBPjκ–KO^TEC^ thymi of the indicated ages. Data are shown as mean percentages ± SEM, (n ≥ 4). **(E)** Percentages of B, NK and myeloid cells among total thymus cells from 12-month-old WT *Foxn1^+/+^x RBPjk^fl/fl^* or RBPjκ–KO^TEC^ mice. Data show mean percentages ± SEM, (n ≥ 4). **(F)** Percentages of either total Thy1^+^ T- and Thy1^-^ non-T-lineage cells (left) or DN, DP and CD4^+^ and CD8^+^ SP thymocytes (right) reconstituting the thymus of WT C57BL/6J (Ly5.1^+^) mice transplanted with BM cells from WT *Foxn1^+/+^x RBPjk^fl/fl^* or RBPjκ–KO^TEC^ (Ly5.2^+^) mice. Data are shown as mean percentages ± SEM, (n=3). *p<0.05; **p<0.01; ***p<0.001. p values were calculated using a two-tailed t-test ns, not significant.

The above findings showing a reduced and disorganized medulla in RBPjκ-KO^TEC^ postnatal thymi is consistent with the possibility that specific abrogation of Notch activation in TECs results in a premature thymic involution and leads to an impaired thymus function. To investigate this possibility, we analyzed T-cell development and thymic output in RBPjκ-KO^TEC^ and WT *Foxn1^+/+^x RBPjk^fl/fl^* aged mice by flow cytometry. We found that thymocyte numbers were equivalent in young WT and RBPjκ-KO^TEC^ mice (not shown), but decreased significantly in mutant compared to WT mice along life, to up to 70% by 12-months (**Figure 7A**). The observed thymocyte decrease paralleled a weak but significant reduction of the CD4^+^CD8^+^ double positive (DP) thymocyte subset in RBPjκ-KO^TEC^ mice (**Figures 7B, C**). This decrease could be attributed to a homeostatic defect in mTECs (18) and associated paracrine signaling axes (46), which may indirectly affect cortical epithelial cell function. Alternatively, it may directly result from a defective function of cTECs in mutant mice. In addition to the DP cell loss, we observed a marked increase of non-T lineage (Thy1^-^) cells in mutant mice compared to WT littermates, which accounted for up to 20% of total thymic cells at 12-months (**Figure 7D**). Flow cytometry analyses using lineage-specific markers identified B cells as the major non-T cell type accumulating in the adult mutant thymus, but NK cells and myeloid cells were also significantly increased (**Figure 7E**). As increased frequencies of thymic B cells is a feature associated with thymic involution in aged mice (18), our results suggest that a defective thymic microenvironment rather than an intrinsic functional defect of developing thymocytes is responsible for the observed expansion of non-T lineage cells in RBPjκ-KO^TEC^ thymi. To assess this possibility, we performed adoptive transfer experiments consisting on intra-venous injection of total hematopoietic cells isolated from the BM of either RBPjκ-KO^TEC^ or WT *Foxn1^+/+^x RBPjk^fl/fl^* Ly5.2^+^ littermates into lethally-irradiated C57BL/6J Ly5.1^+^ normal mice. Flow cytometry analyses of cells recovered from the thymus of host mice at 4 months post-transplant revealed no differences in the reconstitution efficiency of BM progenitors from either WT or mutant mice, as indicated by the equivalent proportions of Thy1^+^ T-lineage cells and DP, double negative (DN) and single positive (SP) subsets present in the host thymi (**Figure 7F**). Therefore, we can exclude an intrinsic functional defect of T-cell progenitors derived from RBPjκ-KO^TEC^ mutant mice. Based on our results, we concluded that *Foxn1*-controlled impaired activation of canonical Notch signaling leads to an accelerated loss of mTECs accompanied by disruption of the medulla integrity in the postnatal thymus, which concurs with an aberrant increase in the proportion of thymic non-T lineage cells and a decrease in DP thymocyte numbers, compatible with a premature thymic involution.

## Discussion

We have studied the potential contribution of the Notch pathway to postnatal TEC biology using two complementary strategies. First, we analyzed Notch activation *in situ* in the human postnatal thymus by performing quantitative immunohistochemistry and confocal imaging. Our results show for the first time that Notch activation is regulated *in vivo* in the human thymic epithelium in a spatio-temporal manner. We found that Notch signaling, mediated in particular through the Notch1 receptor, is induced *in situ* in postnatal human TECs mostly located at the medulla, and this activation pattern is conserved in the mouse. Importantly, numbers of mTECs showing Notch activation increase significantly with age in both human and mouse postnatal thymi, suggesting a conserved role for Notch signaling in TEC homeostasis during aging. To further investigate this possibility, we made use of an *in vivo* genetic model of *Foxn1*-controlled conditional inactivation of Notch signaling in murine epithelial cells. The model revealed that impaired Notch signaling in mutant TECs leads to an accelerated age-dependent decrease of postnatal mTECs that results in the disruption of the medullary thymic microenvironment and in an accelerated thymus atrophy.

The observation that Notch signaling is activated *in situ* in the epithelial compartment of the postnatal thymus was somehow unexpected, as preliminary studies in mice (35–37), recently confirmed by genetic approaches, pointed to a role of Notch signaling limited to embryonic stages of TEC development, while Notch activation has been shown to be downregulated afterwards disappearing in postnatal TECs (38, 39). Accordingly, Notch signaling critically regulates mTEC-lineage fate specification of embryonic TEC progenitors, but further mTEC development is dependent on repression of Notch activation (39), a process that may rely on HDAC3 function (37). These results seem in conflict with our finding that Notch is active *in vivo* in postnatal TECs; particularly, in a significant population of TECs located at the medulla. However, an important question is whether such mTECs with active Notch are immature or fully mature mTECs. While our current results cannot give a definitive answer to this question, the first possibility seems very likely considering that, during embryonic TEC development, Notch signaling is critical not only for mTEC specification, but also for maintenance/expansion of the pool of undifferentiated TEPC and mTEC-restricted progenitors (38, 39). Considering that both TEPC and mTEC progenitors have been identified in the adult murine thymus (23–27), an attractive explanation for our results would be that expression of active Notch in the postnatal thymus is restricted to the TEPC and/or mTEC progenitor pools (50), thus controlling the high turnover of mTECs and their maintenance and regeneration in the adult thymus (17). In fact, it is known that the TEC compartment has an extensive cell division in fetal and neonatal life, but postnatal TEC proliferation decreases significantly by 4 weeks (17), while medullary TECs display relatively high turnover rates also during the postnatal stage. An alternative possibility is supported by the finding that mTECs that display active Notch signaling accumulate in the postnatal human thymus in HC, a structure derived from terminally differentiated mTECs, suggesting that Notch activation could be induced in mature mTECs.

Considering the high developmental and functional heterogeneity revealed for the TEC compartment (14, 51), generation of conclusive results on the exact maturation stage of postnatal mTECs that activate Notch signaling *in vivo* demands further studies. Nonetheless, an interesting finding of our work is that postnatal mTECs activate Notch signaling in an age-dependent manner in both humans and mice, as revealed by quantitative analyses. Comprehensive kinetics in mice showed that numbers of mTECs with active Notch signaling increased by 3-months of age, immediately after achievement of maximal thymic cellularity and coincident with the initiation of thymic involution (17). It is thus possible that activation of Notch signaling is upregulated at early postnatal ages to counteract the loss of mTECs associated with thymic involution (17). Supporting such a role, our loss-of-function genetic approach has shown that abrogation of canonical Notch signaling results in decreased proportions of TECs, mostly of mTECs, during postnatal life, while normal mTEC numbers were found during the first month of life. These findings concur with the results shown by Blackburn and coworkers using a distinct Foxn1-Cre x Rbpjfl/fl mouse model (38), in which mTEC generation is impaired in embryonic life, but mTECs proportions were normalized at week 8 after birth. Given that transgenic Cre expression in FoxN1-Cre mice parallels endogenous Foxn1 expression in epithelial cells (E11.5), and Foxn1-controlled expression is induced one day later as indicated by β−galactosidase expression (42), Notch signaling could not be abolished before E12.5 in mutant mice, which corresponds to a time in development when TEC progenitors have been established and their progeny has contributed to an initial thymus primordium. Thus, a relatively late timing of RBP-Jκ deletion could result in reduced numbers rather than total loss of mTEC progenitors that would be able to recover normal numbers of mTECs in mutant thymi early after birth. Importantly, we show that, after mTEC numbers are normalized, abrogation of Notch signaling in mutant mice results in a further age-dependent dramatic loss of mTECs. Whether mTEC loss results from the impaired maintenance/expansion or the enhanced mortality of mTECs and/or mTEC progenitors remains to be determined; but itconcurred with a marked disorganization of the thymic medulla architecture, and a significant reduction in thymus size, together with diminished thymocyte numbers, decreased proportions of DP thymocytes and the accumulation of intrathymic B cells. As all these features are associated with age-dependent thymic involution (16–18), we concluded that abrogation of Notch signaling in postnatal TECs may accelerate thymus aging and impaired thymus function (49). Accordingly, DP thymocyte frequency is a readout of thymus functionality that correlates inversely with thymus involution and mTEC loss (18), and has been associated with apoptosis susceptibility of thymocytes (52). Although we cannot establish whether DP thymocyte loss is directly dependent on the homeostatic defect in mTECs, it is posible that defective mTECs located at the corticomedullary junction, where accumulation of ICN1^+^ TECs was observed, could impact viability of recently selected DP thymocytes migrating from the cortex to the medulla. Alternatively, a defective mTEC paracrine signaling axis may indirectly affect cortical epithelial cell function (46), or defective DLL4 expression on mutant cTECs (53) may affect DP thymocyte generation.

Understanding how mTEC maintenance and regeneration are regulated in the adult thymus downstream of Notch signaling is of critical relevance for understanding thymic involution, but the effectors involved in such Notch-mediated function remain to be identified. In this regard, it is worth noting that Myc and cyclin D1, two well-known downstream targets of Notch signaling have been shown to contribute to TEC growth and to promote a dramatic increase of thymus size upon ectopic expression in TECs (18, 54, 55). Notably, as described for Notch activation (38, 39) Myc transcription declines in TECs during embryonic development, and minimal levels have been described after birth, suggesting that regulation of Myc function is required to limit thymic growth in adult mice. As Myc expression in adult TECs drives proliferation and results in thymic regeneration (54), it is possible that Notch signaling controls mTEC maintenance and thymic involution through Myc. An important question is how Notch signaling is temporally regulated to control mTEC maintenance and thymus homeostasis. To answer this question, we have to consider that spatio-temporal regulation of Notch ligand expression defines particular Notch signaling microenvironments in the thymus (40, 56). Manley and coworkers have shown tan Notch1 signaling in TEC development begins soon after the onset of Foxn1 expression, when Jag1 and DL4 Notch ligands are expressed (39). Notch1 could also be the receptor mediating Notch signaling in postnatal mTECs, given the coincident patterns of Hes1 and active intracellular Notch1 (ICN1) expression observed in both human and mouse postnatal thymi. Although we cannot ignore the expression of Notch3 in human mTECs, this receptor could be upregulated following Notch1-mediated signaling as reported in thymocytes (56). In the postnatal thymus medulla, a possible source of Notch1 ligand would be other mTECs, which express Jag1 (40), though Notch ligand presented by developing thymocytes could induce Notch1 activation as well. In this regard, it is important to note that crosstalk between developing thymocytes and TECs in one of the mechanisms that control TEC development and likely thymus involution (16–18). TECs depend on the presence of thymocytes for their differentiation and organization (57, 58), and they reciprocally provide the signals that regulate T lymphocyte generation (59). Therefore, Notch activation could be negatively regulated in mTECs during thymopoiesis once a given cellular density of SP thymocytes has been reached at the medulla. In this regard, recent results by Blackburn’s group provided evidence of a cross-regulatory relationship between Notch and Foxn1, the master regulator of TEC differentiation that is required to maintain the postnatal thymic microenvironment in a dosage-sensitive manner (60–62), suggesting a Foxn1-mediated repression of Notch activity that could be reinforced *via* its direct ligands (38). Conversely, Foxn1 downregulation during thymus involution (60–62) could trigger Notch activation to counteract mTEC loss and thymus aging. While further studies are required to reach a full understanding of mechanisms controlling postnatal mTEC turnover and thymic involution, our results point toward manipulation of Notch signaling as a novel and promising strategy for thymus regeneration during aging.

## Data Availability Statement

The original contributions presented in the study are included in the article/**Supplementary Material**. Further inquiries can be directed to the corresponding author.

## Ethics Statement

The studies involving human participants were reviewed and approved by Spanish National Research Council Bioethics Committee. Written informed consent to participate in this study was provided by the participants’ legal guardian/next of kin. The animal study was reviewed and approved by Animal Experimentation Ethics Committee of the Comunidad de Madrid (PROEX 002.16/21).

## Author contributions

MLT conceptualized, designed and supervised the study, wrote the manuscript and acquired funding. MG-L, MM, CC, and JA collected and processed the samples, performed the experiments, analyzed the data, and prepared the figures. SŽ and GH developed the animal models and supervised the study. MG-L drafted the first version of the manuscript. All authors contributed to the article and approved the submitted version.

## Funding

This work has been supported by the European Union Seventh Framework Programme (FP7/2007-2013) collaborative project ThymiStem (602587 to MLT) and by Spanish Ministry of Science and Innovation. (Agencia Estatal de Investigacion/European Regional Development Fund, European Union, SAF2014-62233-EXP, SAF2016-75442-R and PID2019-105623RB-I00 to MLT). Institutional grants from the Fundación Ramón Areces and Banco de Santander to the Centro de Biología Molecular Severo Ochoa are also acknowledged.

## Conflict of Interest

The authors declare that the research was conducted in the absence of any commercial or financial relationships that could be construed as a potential conflict of interest.

## Publisher’s Note

All claims expressed in this article are solely those of the authors and do not necessarily represent those of their affiliated organizations, or those of the publisher, the editors and the reviewers. Any product that may be evaluated in this article, or claim that may be made by its manufacturer, is not guaranteed or endorsed by the publisher.
