# Supplementary material for: Abrogation of Notch Signaling in Embryonic TECs Impacts Postnatal mTEC Homeostasis and Thymic Involution
Source: Front Immunol. 2022 May 30;13:867302. doi: 10.3389/fimmu.2022.867302 (PMC9189879; doi:10.3389/fimmu.2022.867302)
Supplement: Supplementary file 1 [file DataSheet_1.pdf]

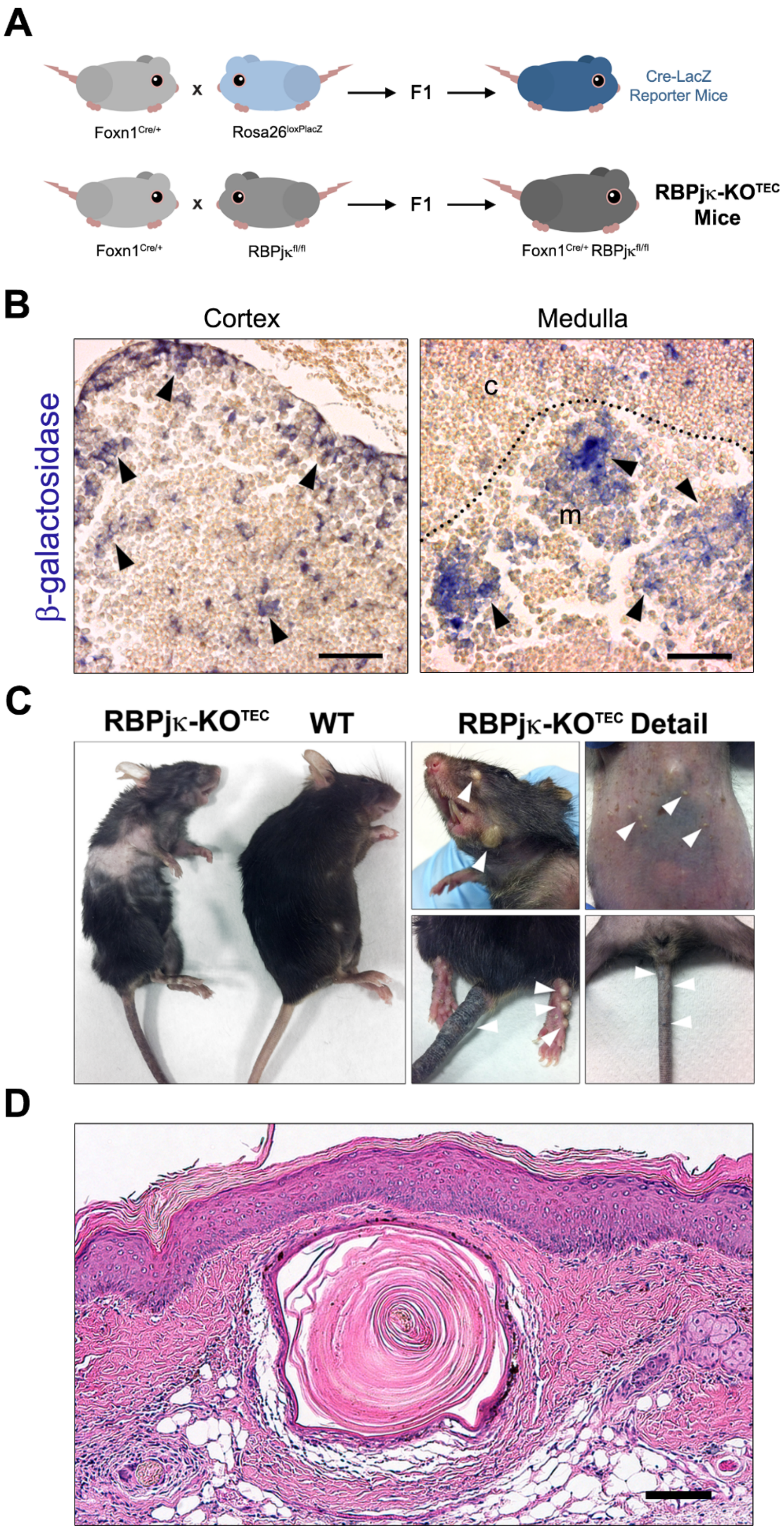

Supplementary Figure 1. **Murine model of *Foxn1*-controlled abrogation of Notch signaling in epithelial cells. A)** Scheme showing mouse breeding strategies leading to the generation of Cre-LacZ reporter and RBPjκ-KO^{TEC} mouse lines used in this study. Cre-LacZ reporter mice were generated by crossing Foxn1-Cre mice to the *Rosa26^{loxPlacZ}* reporter strain (top). Conditional mice with impaired activation of canonical Notch signaling in epithelial cells (RBPjκ-KO^{TEC}) were obtained by crossing Foxn1-Cre mice to the *Rbpj$^{fl/fl}$* conditional knockout mouse line (bottom). **B)** β-galactosidase staining of thymus cortex (left) and medulla (right) from neonatal (3-days) Cre-LacZ reporter mice. Arrowheads indicate β-galactosidase staining (blue) in TECs, confirming TEC-specific Cre-mediated recombination in the thymus. Dotted line: corticomedullary junction. Scale bar: 200μm. Images are representative of at least five images per sample (n = 3). **C)** Macroscopic inspection of WT and RBPjκ-KO^{TEC} mice showing cutaneous lesions at the face, footpad, tail and dorsal and ventral skin of 8-month-old mutant mice. **D)** Hematoxylin and eosin histological section of footpad skin tissue from a 9-month-old RBPjκ-KO^{TEC} mouse showing keratin cysts. Scale bar: 100μm.

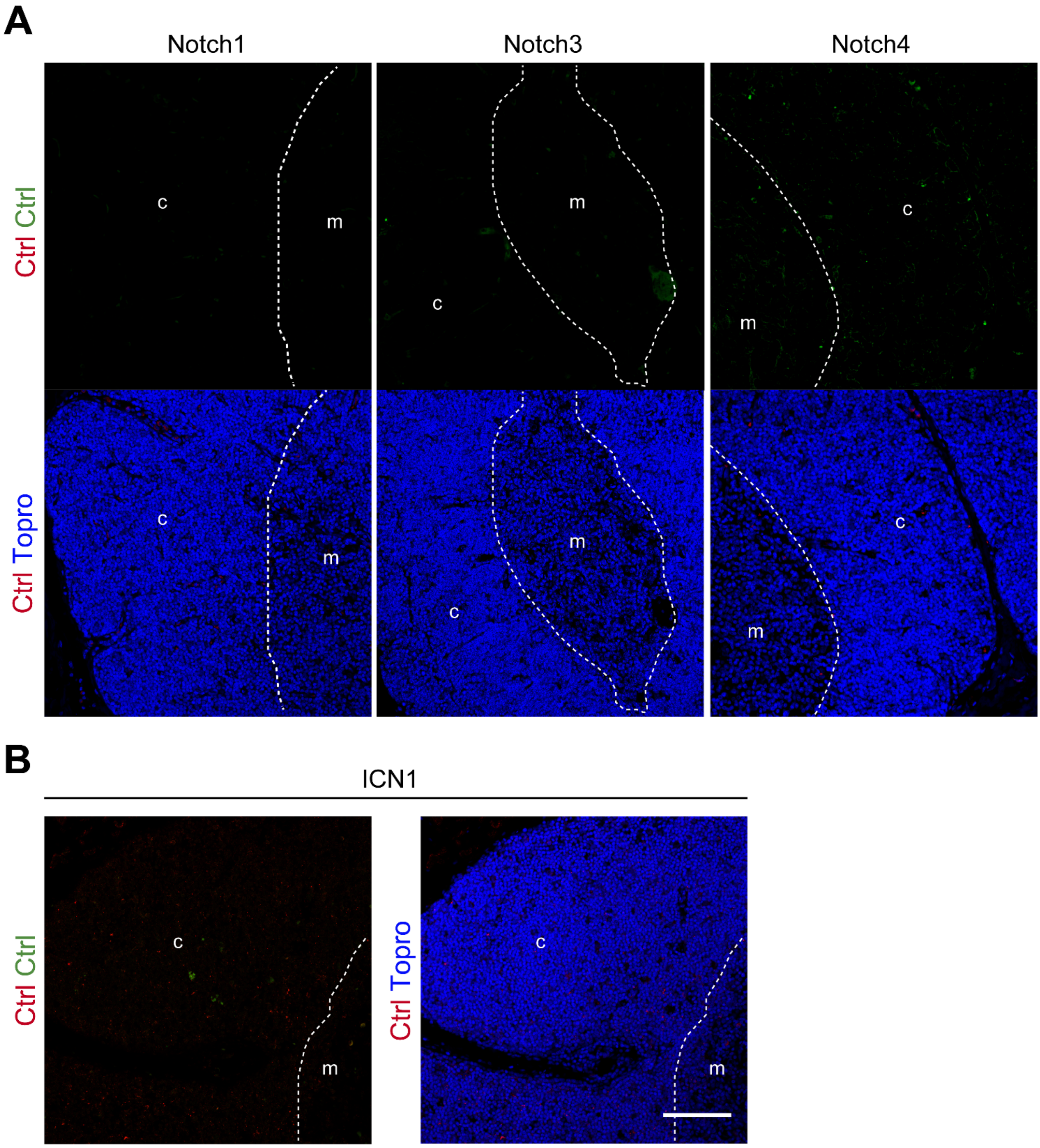

Supplementary Figure 2. Immunohistochemistry controls. **A, B)** Background staining of Notch receptors and pCK (A), or active Notch1 (ICN1) (B) in human thymus sections, defined by isotype-matched controls. Dotted line: corticomedulary junction; c: cortex; m: medulla. Scale bar: 100µm.

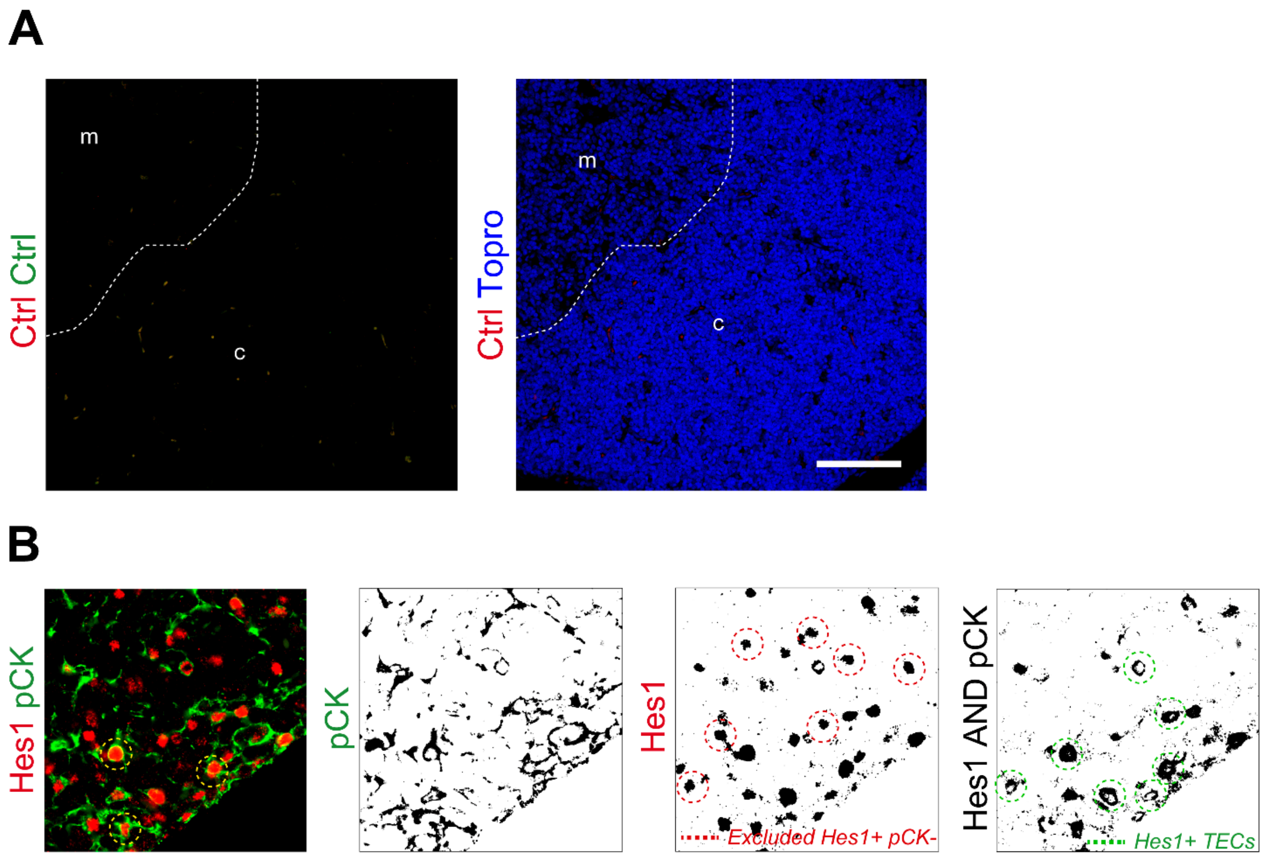

Supplementary Figure 3. Quantitative immunohistochemistry approaches. **A)** Background staining of Hes1 and pCK on human thymus sections defined by isotype-matched controls. Dotted line: corticomedulary junction; c: cortex; m: medulla. Scale bar: 100µm. **B)** Quantification strategy of Hes1^{+} (and ICN1^{+}) nuclei measurements per pCK^{+} ROIs defined in the cortex and the medulla. Left image shows the starting image (with no Topro stainning). Next image shows the binary pCK channel after pCK^{+} area selection by thresholding that was used to create a panCK ROI, which allowed calculation of the total TEC area per field (in µm2). The third image shows the binary Hes1 channel after Hes1^{+} area selection by thresholding, and the image on the right shows the result of applying the logical operator "AND" to both pCK and Hes1 binary images. From this image, non-epithelial Hes1^{+} cells (red circles) are excluded. Only signal appearing in both Hes1^{+} and pCK^{+} binary images is shown (green circles).

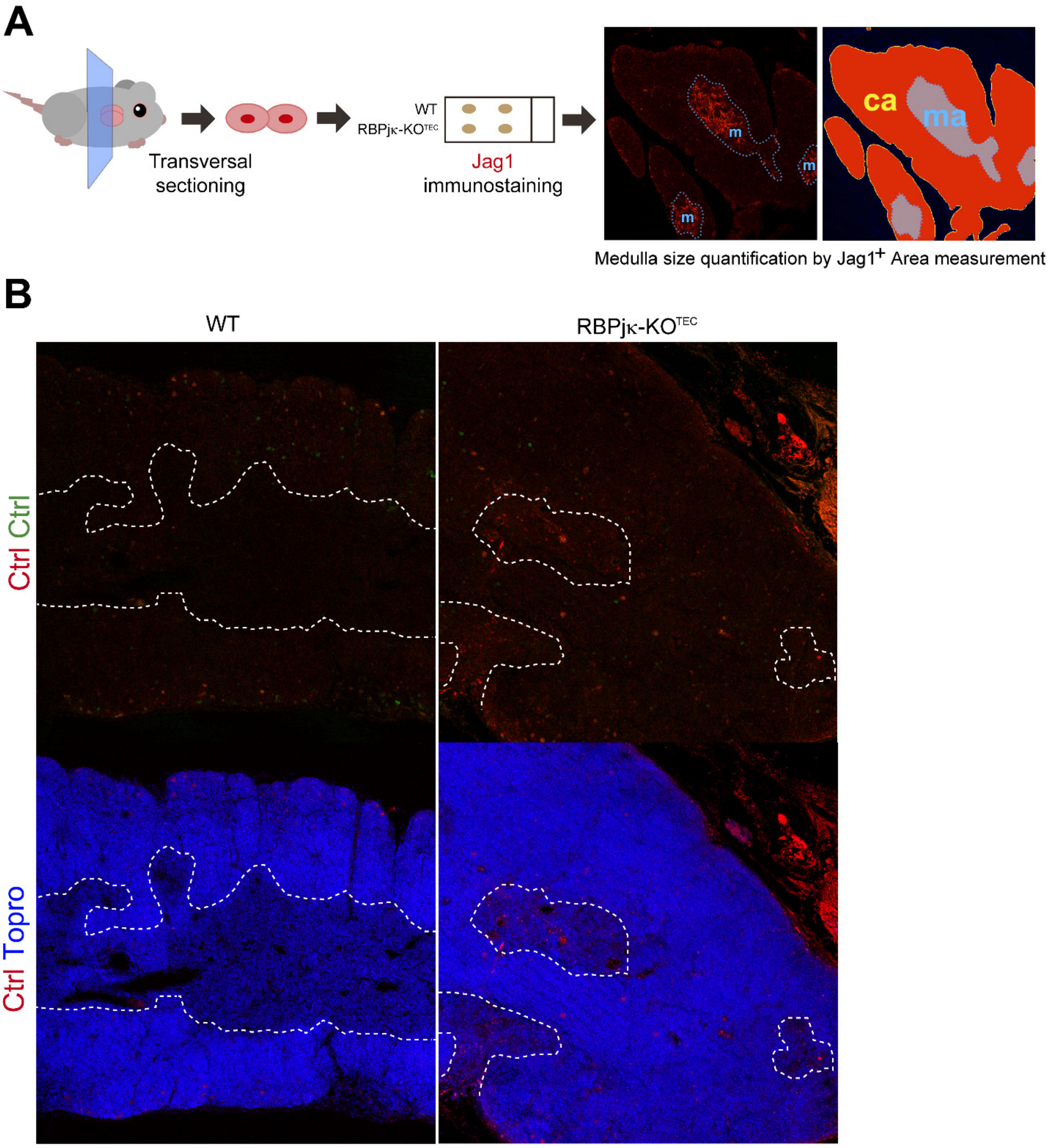

Supplementary Figure 4. **Histomorphometric analysis of mouse thymus. A)** Infograph of the histomorphometric analysis of thymus medulla. Transverse sections obtained from formalin-fixed paraffin-embed WT or RBPjκ-KO^{TEC} thymus samples were stained with antibodies against Jag1 (expressed exclusively on TECs located at the medulla) and anti-pCK (expressed on cTECs and mTECs). Cortical area (ca) and medullary area (ma) were calculated based on Jag1-stained area and total thymic area (pCK and Topro3 staining). Dotted line: corticomedulary junction; m: medulla. **B)** Background staining of Jag1 and pCK on WT or RBPjκ-KO^{TEC} mouse thymus sections defined by isotype-matched controls. Dotted line: corticomedulary junction. Scale bar: 100µm.

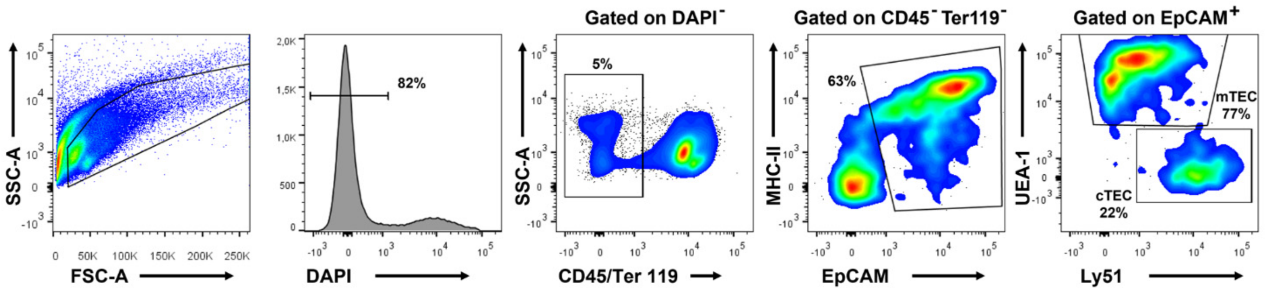

Supplementary Figure 5. Sequential gating strategy for flow cytometry analysis of murine mTECs and cTECs. Cell suspensions obtained by collagenase dissociation of postnatal mouse thymi were labelled with DAPI to exclude death cells ($DAPI^+$) from the analysis. Alive hematopoietic and erythroid-lineage cells identified by CD45 or Ter119 expression, respectively, were subsequently excluded. $CD45^-$ $Ter119^-$ thymic cells expressing EpCAM and either low or high MHC classII antigens were then electronically gated and identified as TECs. Expression of UEA1 mTEC marker and Ly51 cTEC marker was assessed on $EpCAM^+$-gated TECs.

Supplementary Table 1. Antibodies used in immunohistochemistry.

Specificity	Reactivity	Clone	Host species	Supplier	Conjugate
Anti-mouse IgG1	M	Polyclonal	Goat	Thermo	Alexa-488
Anti-mouse IgG2a	M	Polyclonal	Donkey	Thermo	Alexa-555
Anti-Rabbit IgG	Rbb	Polyclonal	Goat	DAKO	HRP
Anti-Rabbit IgG	Rbb	Polyclonal	Goat	Vector Lab.	Biotin
Anti-Rabbit IgG	Rbb	Polyclonal	Donkey	Thermo	Alexa-488
Cleaved Notch1	H, M	D3B8	Rabbit IgG	Cell Sign.	Unconjugated
Hes1	H, M	D6P2U	Rabbit IgG	Cell Sign.	Unconjugated
Jag1	H, M	28H8	Rabbit IgG	Cell Sign.	Unconjugated
Notch1 (C-20)	H	Polyclonal	Rabbit IgG	Sta. Cruz	Unconjugated
Notch3	H	Polyclonal	Rabbit IgG	Abcam	Unconjugated
Notch4	H	Polyclonal	Rabbit IgG	Abcam	Unconjugated
Pan-cytokeratin	H, M, Rbb	Polyclonal mixture	Mouse IgG1 and IgG2a	SIGMA	Unconjugated

H: human; M: mouse; Rbb: rabbit.

Supplementary Material

Genotyping of mutant mice. Selection of *Foxn1*$^{Cre/+}$*x RBPjκ*$^{fl/fl}$ mice was performed by PCR genotyping of genomic DNA obtained by proteinase K (Sigma) digestion of 3 weeks-old mouse ear discs tissue. Primers used to detect Cre recombinase transgene were P1, 5'-TCT GAT GAA GTC AGG AAG AAC C-3', and P2, 5'-GAG ATG TCC TTC ACT CTG ATT C-3', which generates a fragment of about 500bp long. PCR was performed for 34 cycles of 94ºC (30 sec), 58ºC (40 sec), and 72ºC (1 min). For the detection of the RBPjk$^{fl/fl}$ alleles the following primers were used: P3, 5'-ACC AGA ATC TGT TTG TTA TTT GCA TTA CTG-3', and P4, 5'-ATG TAC ATT TTG TAC TCA CAG AGA TGG ATG-3', which generates a product of 430bp long for the RBPjk$^{fl/fl}$ allele and 274bp long for the WT allele. For specific detection of RBPjκ deletion, the primer P5, 5'-TAA TGC ACA CAA GCA TTG TCT GAG TTC-3' was used in combination with primer P3 which yields no product in a non-deleted allele or a 640bp long product when the RBPjk gene has been successfully deleted. In this case, PCR was performed for 40 cycles of 94ºC (30 sec), 60ºC (1 min) and 72ºC (1 min). PCR products were subjected to agarose electrophoresis and visualized with ethidium bromide.
